# Surface Assessment *via* Grid Evaluation (SuAVE) for Every Surface Curvature and Cavity Shape

**DOI:** 10.1021/acs.jcim.2c00673

**Published:** 2022-08-10

**Authors:** Denys E. S. Santos, Kaline Coutinho, Thereza A. Soares

**Affiliations:** † Departamento de Química Fundamental, Universidade Federal de Pernambuco, Cidade Universitária, Recife 50740-560, Brazil; ‡ Instituto de Física, Universidade de São Paulo, Cidade Universitária, São Paulo 05508-090, Brazil; § Hylleraas Centre for Quantum Molecular Sciences, University of Oslo, 0315 Oslo, Norway

## Abstract

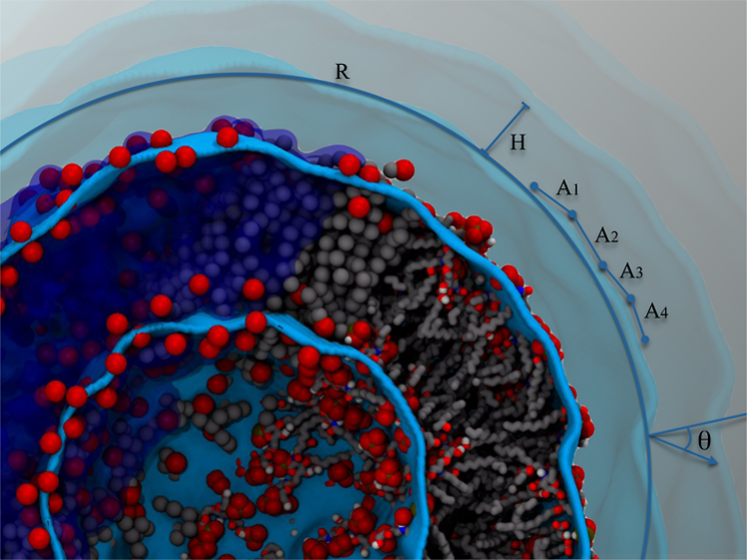

The surface assessment *via* grid evaluation (SuAVE) software was developed to account for the effect of curvature in the calculations of structural properties of chemical interfaces regardless of the chemical composition, asymmetry, and level of atom coarseness. It employs differential geometry techniques, enabling the representation of chemical surfaces as fully differentiable. In this article, we present novel developments of SuAVE to treat closed surfaces and complex cavity shapes. These developments expand the repertoire of curvature-dependent analyses already available in the previous version of SuAVE (*e.g.*, area per lipid, density profiles, membrane thickness, deuterium-order parameters, volume per lipid, and surface curvature angle) to include new functionalities applicable to soft matter (*e.g.*, sphericity, average radius, principal moment of inertia, and roundness) and crystalline porous materials (*e.g.*, pore diameter, internal void volume, total area, and the total void volume of the unit cell structure). SuAVE can accurately handle chemical systems with high and low atom density as demonstrated for two distinct chemical systems: the lipid A vesicle and a set of selected metal–organic frameworks. The SuAVE software v2.0 is fully parallel and benefits from a compiler that supports OpenMP. SuAVE is freely available from https://github.com/SuAVE-Software/source and https://www.biomatsite.net/.

## Introduction

In the last years, a few computational tools have been developed to perform structural analysis of membrane simulations.^1−9^ Most of these tools employ either Voronoi tessellations or Delaunay triangulations to estimate the area per lipid and other structurally relevant properties. These algorithms cannot be directly used to generate smooth and continuous surfaces and thus require the development and implementation of different approximations with varying degrees of accuracy. Voronoi-based algorithms rely on the projection of the 3D coordinates of the chemical system onto a 2D grid through domain tessellation of the 2D lattice to assign properties to each polygon corresponding to a lipid molecule.^1,4^ In some cases, Voronoi tessellations are computed through the projection of the membrane onto the 2D plane.^5,7^ GridMAT-MD was one of the first software developed to analyze membrane properties such as area per lipid (*A*_L_) and bilayer thickness (*D*_HH_) which would be expressed as a two-dimensional contour plot of membrane thickness and a polygon-based tessellation of the individual lipid headgroups.^1^ Later, computational tools were added to the repertoire of membrane-specific properties that could be calculated (*e.g.*, deuterium-order parameter (*S*_CD_), lipid mixing/demixing entropy, transmembrane voltage, and head group orientation).^1,4,5,7^ These tools were better suited to treat flat bilayers than membranes undergoing curvature changes associated with a wide range of biological phenomena.

Recently, this limitation was sought to be addressed by three computational tools.^2,3,8^ FATSLiM applies a surface reconstruction algorithm based on the calculation of local normal vectors along the membrane surface from which tangent Voronoi polygons are projected.^3^ In contrast to previous Voronoi-based tools, FATSLiM computes the 2D projections locally for each 3D position instead of projecting the lipid head groups onto a single 2D plane. Each plane then comprises a Voronoi polygon from which structural and physical properties can be estimated. For this reason, FATSLiM can be employed to analyze the structural properties of flat and curved membranes and vesicles. However, as adverted by Bhatia and co-workers,^2^ the local Voronoi tessellation approach can overestimate the area per lipid when the Voronoi cells of adjacent atoms do not share edges. This could happen, for instance, when the interaction pattern of lipids composing the membrane yields holes in the surface, which is often the case in regions of high curvature. One step ahead, MemSurfer employed an approach based on Delaunay triangulations and a discrete conformal parameterization of the membrane surface to compute the required triangulation with minimal distortions to neighborhoods of lipids.^2^ This approach provides a better approximation for curved surfaces because the incident Delaunay triangles can decompose the local neighborhood onto different planes in contrast to a single plane in the Voronoi cell. Therefore, it minimizes the main issue with Voronoi-based tools, which is the disposal of *z*-coordinates when projecting 3D points onto the 2D plane. However, the discrete conformal surface parameterization implemented in MemSurfer cannot be currently applied to closed topologies such as vesicles.^2^ Furthermore, the discontinuous surfaces generated by Delaunay triangulations are susceptible to the appearance of singularities (*i.e.*, boundaries, sharp features, and nonmanifolds) in the computed surface that may lead to failure of meshing algorithms and imprecise calculation of molecular areas or volumes for instance.

The third computational tool to compute highly curved surfaces is surface assessment *via* grid evaluation (SuAVE) which makes use of radial Gaussian functions to interpolate atom positions scattered across interfaces of any shape *via* the direct employment of well-established differential and computational geometry techniques.^8^ The main advantage of this method is the generation of smooth surfaces that are continuous and, therefore, fully differentiable. In contrast to Voronoi/Delaunay-based algorithms, 3D coordinates of the chemical system are projected onto a 3D grid with *z*-coordinates obtained by an average over Gaussian functions built for each atom position in the chemical surface. After the computation of the projected 3D surface, a set of simple numerical algorithms can be employed to calculate a wide range of properties from a single structure or trajectories at the atomistic or coarse grain level. Several curvature-dependent properties can be calculated by SuAVE: area per lipid (*A*_L_), density profiles (ρ(*z*)), membrane thickness (*D*_HH_), curvature order parameters (*S*_C_), and volume per lipid (*V*_L_), as well as vesicle total area (*A*_g_), volume (*V*_g_), mean radius (*R*_a_), sphericity (Ψ_g_), and radius of gyration. Furthermore, it can handle a variety of chemical surfaces, whether soft-matter, crystalline, or liquid/liquid/gas interfaces (see ref (8) for details).

The interpolation function implemented in SuAVE is general and applicable to open surfaces, whether flat or curved, as previously shown for lamellar and micellar systems.^8^ In this code, a uniform grid is generated in the *xy*-plane and the *z*-coordinate of the 3D grid surface is adjusted by a fitting procedure. The grid points are defined by Cartesian coordinates  and the expression for the fitting is given by
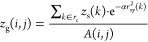
1
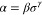
2where *z*_s_(*k*) is the *z*-coordinate of the *k* atom that compose the chemical surface,  is the *xy*-distance between a given grid point (*i*,*j*) and the *k* atom, α is the width of the Gaussian distribution that is related to the atomic density of the chemical surface σ using an empirical power law obtained to minimize the root-mean-square distance (RMSD) between the grid surface and atomic positions, and  is the weight normalization. A cutoff radius *r*_c_ was implemented to save the computational time. Therefore, the *z*-position of each grid point is obtained as an average over the atomic *z*-coordinate *z*_s_(*k*), weighted by the Gaussian distribution of the *xy*-distance between a given grid point and the surface atoms, that is, surface atoms with a smaller *xy*-distance to the grid point contribute more to the average *z*-position. The values of β and γ were previously parameterized for describing open surfaces (β = 0.0214 and γ = 0.8493).^8^ For the description of closed surfaces, it was necessary to reparametrize and validate β and γ for the accurate interpolation of points across more complex surfaces (see the Supporting Information). The implementation of new functionalities for the analyses of closed surfaces also required the use of spherical coordinates and geodesic distance to identify the closer atoms and define the Gaussian weight contribution of each atom. Furthermore, the surface normal vector is now compared with the canonical radial vector from the spherical coordinate system instead of the *z*-axis of the simulation box previously applied for open surfaces oriented at the *xy*-plane. The current version of SuAVE v2.0 is parallel-processed and benefits from compilers that support OpenMP.^10^ Lastly, several new functionalities relevant to the structural analysis of closed systems were implemented in SuAVE v2.0. These functionalities can be applied to a variety of systems, whether deformable soft-matter vesicles or geometrically intricated porous crystalline materials. In this work, we present the developments in SuAVE v2.0 and demonstrate the usefulness of the novel features through the analysis of two rather distinct chemical systems, namely, a lipid A vesicle and a set of selected crystalline porous materials. SuAVE v2.0 remains agnostic with respect to simulation force fields, levels of model resolution (*i.e.,* atomistic or coarse grain), and levels of theory (*i.e.*, quantum or classical approaches), and most importantly, it can handle accurately systems with high or low atom density (*i.e.,* high-density membranes and low-density porous materials). It requires only the Cartesian coordinates of the system in the universal format PDB as input, whether as a single structure or a collection of structures in a trajectory file. SuAVE v2.0 is a free, open-source piece of software licensed under the GNU General Public License v2 or later, and it is available at https://github.com/SuAVE-Software/source and https://www.biomatsite.net/.

## Grid-Based Algorithm for Closed Surfaces

In the first step, the Cartesian coordinates of all system atoms and a set of atomic indexes that compose the closed interface are read in PDB and index files, respectively, as described previously for SuAVE v1.0.^8^ For systems with two closed interfaces such as vesicles, it is necessary to define two sets of atomic indexes composing the outer leaflet (*S*_up_ with *n*_up_ atoms) and inner leaflet (*S*_down_ with *n*_down_ atoms). The Cartesian coordinates of the *S*_up_ set of atoms  are used to generate the geometric center of the closed surface, , and the average radius (*R*_a_). Additionally, the atomic spherical coordinates are also generated: , where , Δ*x*_s_ = *x*_s_ – *x*_0_, Δ*y*_s_ = *y*_s_ – *y*_0_, Δ*z*_s_ = *z*_s_ – *z*_0_, , and .

The grid points of the closed surface are defined by spherical coordinates  constructed in two straightforward steps. First, the angular distribution of a spherical grid is generated around *C*_0_ considering a specific number of partition bins along the angular coordinates θ_g_ and φ_g_. This is defined by the user with the flag *bin* or, optionally, automatically defined by the SuAVE as a function of the atomic density σ of the surface. The grid points (*i*,*j*) are equally spaced throughout the angular coordinates with intervals of Δθ = Δφ = 2π/bin for 0 ≤ θ_g_ < 2π and 0 ≤ φ_g_ ≤ π. Hence, the angular distribution of the grid points is given by θ_g_(*i*) = Δθ(*i* – 1) and φ_g_(*j*) = Δφ(*j* – 1) with *i* varying from 1 to bin + 1 and *j* varying from 1 to (bin/2) + 1, totalizing *n* points in the grid, *n* = (bin + 1)(bin/2 + 1). Second, the radius value of each angular grid point *r*_g_(*i*,*j*) is adjusted by a fitting procedure that considers the position of the interface atoms within the cutoff radius *r*_c_. The fitting procedure is obtained as an average over atomic distances to the center of the surface *r*_s_(*k*), weighted by the Gaussian distribution of the geodesic distance *d*(*i*,*j*,*k*) between a given grid point (*i*,*j*) and the interface atoms, *d*(*i*,*j*,*k*) = *r*_s_(*k*). ΔΦ(*i*,*j*,*k*), with the central angle defined as 

The expression for the adjusted radius of each grid point in the fitting procedure is written as
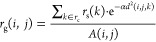
3where  is the weight normalization and α is the width of the Gaussian distribution described by eq 2 with the atomic density of the surface calculated by . A cutoff radius *r*_c_ was previously implemented to optimize the computational time for the calculation.^8^ In SuAVE v2.0, the calculation of this cutoff radius was also adapted to the spherical symmetry. It was defined as the ratio between 3 times the average circumference of the closed surface and the square root of the number of atoms in the closed surface, as presented in the following equation
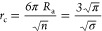
4

**Figure 1:**
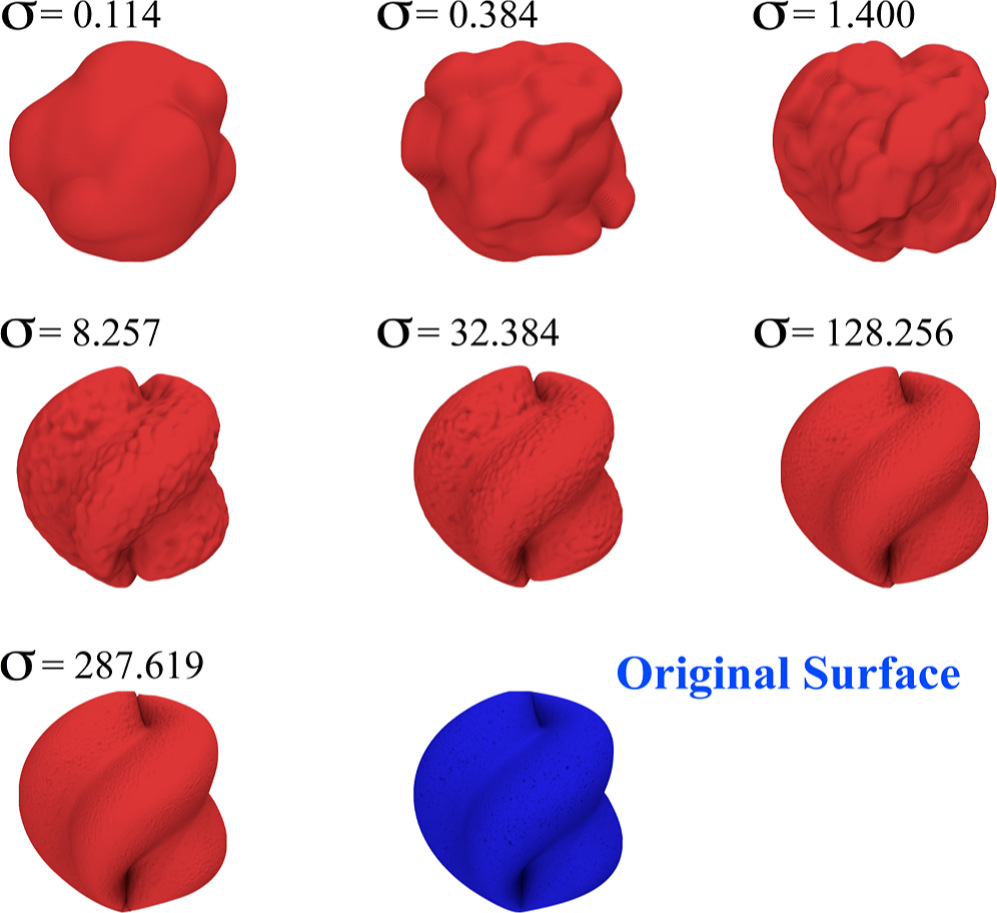
Relation between the surface atomic density (σ) and the fitting of the grid surface. The seven grid surfaces were interpolated using different values of σ taken from the original surface in blue. The parameter σ in this example is expressed in sampling points per nm^2^. For surfaces from real chemical systems, the parameter σ is expressed in atoms per nm^2^.

The Gaussian width α and, consequently, the β and γ parameters (see eq 2), were obtained from a test set of continuous and linearly independent compositions of sinusoidal surfaces (Figure S1 and Tables S1–S5). The α best values were determined to minimize the RMSD between the position coordinates from the fitted grid and the test surfaces (Tables S5). Using this approach, the α parameter can be estimated independently for a collection of surfaces with diverse shapes and in response to the variation of the surface atomic density σ. Then, a best fit between α and σ determined that β = 0.0382 and γ = 0.9968 (Figure S2). This approach to identify the Gaussian width α plays an important role in the grid fitting procedure as the quality of the adjusted surface correlates with the surface atomic density σ used by SuAVE to build the grid surface. In principle, the higher the surface atomic density σ, the better the fitting of the grid surface (Figure 1). However, this is not to be confused with the resolution of the interpolated surface. For instance, several grid surfaces can be built using increasing values of surface atomic densities σ taken from a given original surface (Figure 1). Yet, each one of these fitted surfaces exhibits the same resolution as they contain the same number of grid points. Hence, the tessellation of the surface developed by SuAVE is not limited by the atomic density of the surface σ. The resolution of the interpolated surface is a parameter defined by the user with the flag *bin* which specifies the number of points *n* in the grid. It is limited only by the computational processing times which increase with the number of grid points *n* (see Figure S4).

**Figure 2:**
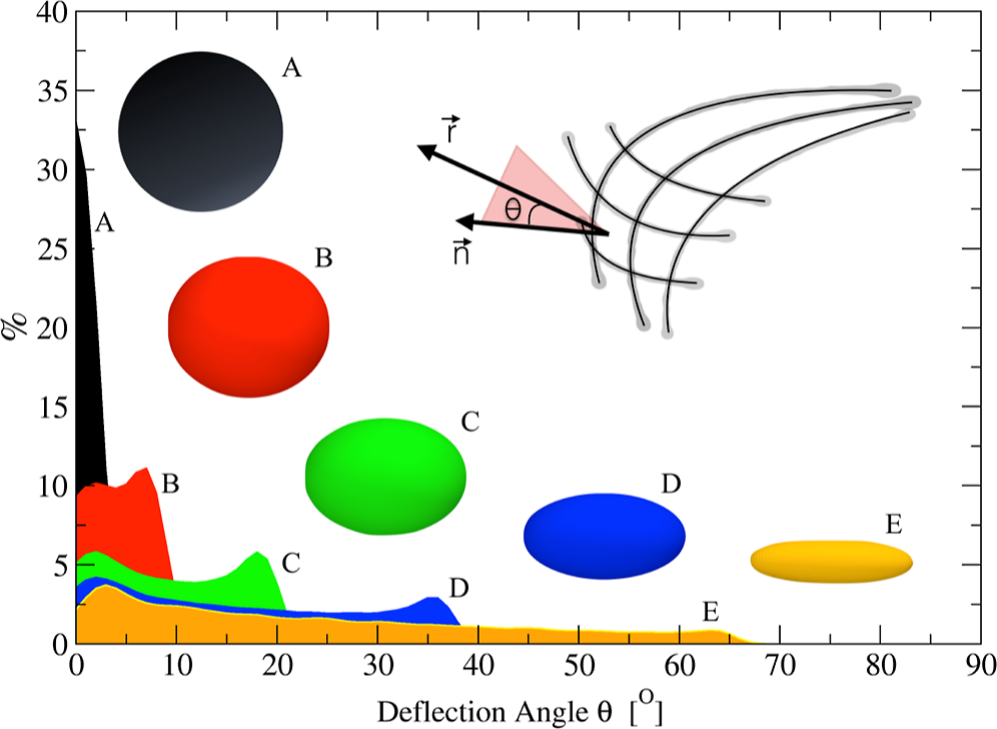
Deflection angle distribution calculated for five ellipsoids presenting distinct eccentricities. The graphical representation of the deflection angle is shown in the inner plot as the angle calculated between the radial vector, *r⃗*, and the surface normal vector, *n⃗*.

After building the grid fitting surface, the geometrical parameters required to characterize the morphology of the chemical interface are calculated. One of these parameters is the deflection angle (θ) formed between the radial vector, *r⃗*, and the surface normal vector, *n⃗*, which is evaluated for each square grid partition of the fitting surface (Figure 2). Each square grid partition is defined by four neighbors points of the grid *G*(*i*,*j*), *G*(*i* + 1,*j*), *G*(*i*,*j* + 1), and *G*(*i* + 1,*j* + 1) (see ref (8) for details). This quantity is then used to calculate the density profiles, distributions of deflection angles, the volume encompassed by the closed surfaces, and the curvature order parameters. An illustration of deflection angle distributions of five different ellipsoids which are ideal representations of morphologies adopted by closed systems is presented in Figure 2. For surfaces close to a spherical shape, the deflection angle distribution presents a maximum near θ = 0°, while for surfaces close to oblate and prolate forms, the distribution is more spread out for θ between 0 and 65°.

## Calculation of Curvature-Dependent Properties for Closed Chemical Surfaces

### Area per Lipid, Average Radius, and Sphericity

The area (*A*_g_), volume (*V*_g_), average radius (*R*_a_), and sphericity (Ψ_g_) of the closed grid surface are calculated by the *s_spher* routine of SuAVE. The total area of the closed grid surface *A*_g_ is calculated by dividing each square grid partition into two spherical triangles, which are treated as two flat triangular partition with vertices defined by grid points {*G*(*i*,*j*), *G*(*i* + 1,*j*), *G*(*i*,*j* + 1)}, and {*G*(*i* + 1,*j* + 1), *G*(*i* + 1,*j*), *G*(*i*,*j* + 1)}. The area of each flat triangular partition  is calculated *via* Heron’s formulae as described for the open surfaces (see ref (8) for details). Then, the area of the closed grid surface is obtained as the sum of all *l* triangular partition areas, *A*_g_ = ∑_*l*_*A*_tp_(*l*).

The volume of the closed grid surface *V*_g_ is also calculated using the flat triangular partitions of the grid to define a triangular-based pyramid whose apex is the center *C*_0_ of the closed surface. Then, the total volume *V*_g_ of a closed grid with average radius, , is obtained as the sum of all triangular-based pyramid volumes defined as
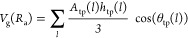
5where  is the height of the pyramid defined as the average radius of the grid points composing the triangular partition and θ_tp_(*l*) is the deflection angle between the surface normal vector *n⃗* and the radial vector *r⃗* of each triangular partition.

The sphericity of closed grid surface Ψ_g_ is calculated as the ratio of the surface area that has the same volume *V*_g_ and the grid surface area itself *A*_g_ using the following expression
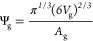
6

**Figure 3:**
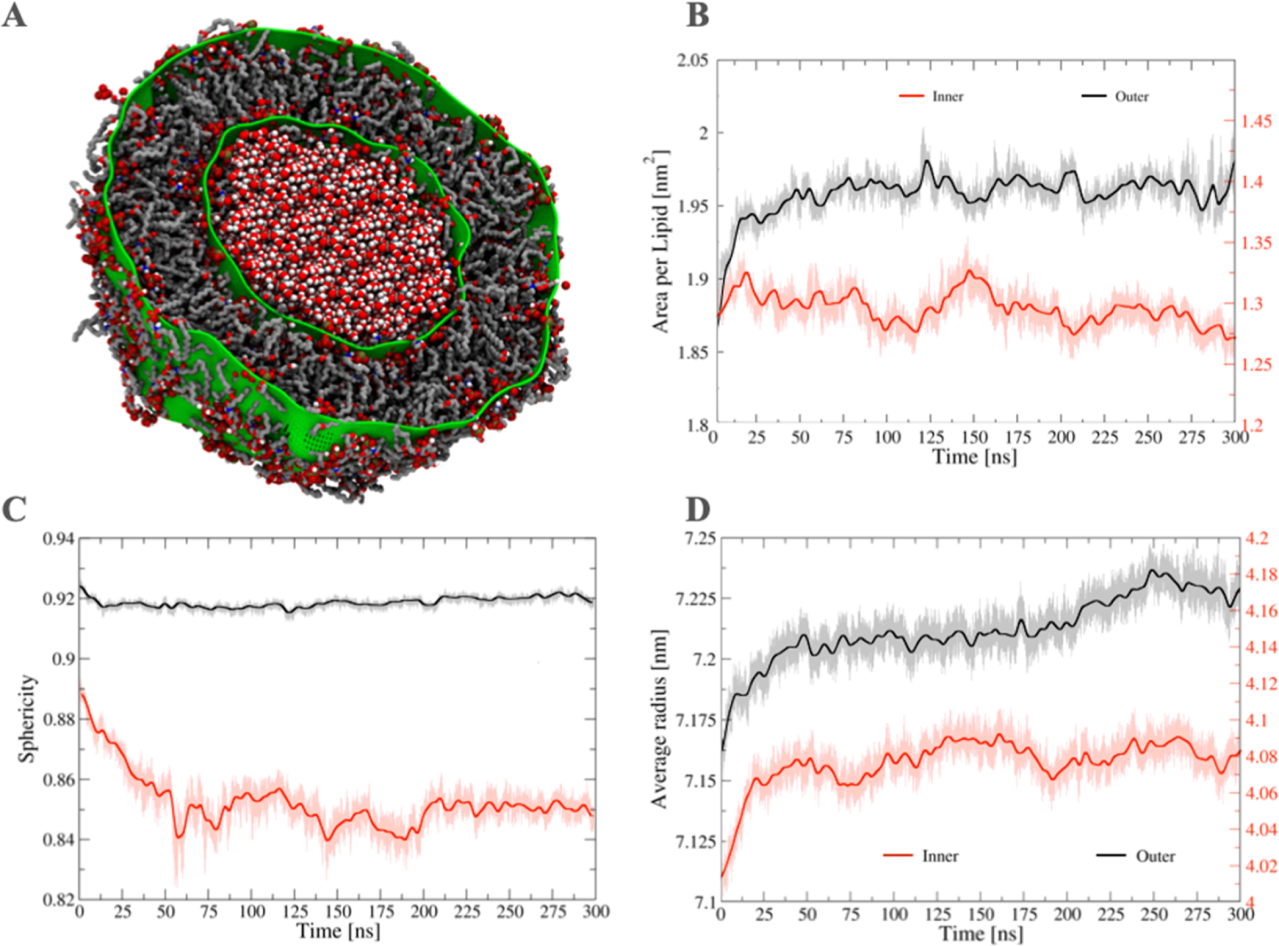
Cross section, area per lipid, sphericity, and average radius calculated for the inner and outer leaflet of a lipid A vesicle. (A) Color representations of the cross section of the lipid A vesicle are the inner and outer fitted grid surfaces (green), the phosphate groups (red), the acyl chains (gray), and the oxygen and hydrogen atoms of the confined water molecules inside the inner surface of the vesicle (red and white, respectively). (B–D) Discrete Fourier transformation (thick lines) was applied to improve the signal-to- noise ratio of the data (thin lines) by using the *s_filter* routine of the SuAVE code. Values of *A*_L_ are consistent with those obtained from lipopolysaccharide bilayer simulations.^11−15^

For a perfect spherical shape, Ψ_g_ is equal to 1, whereas smaller values represent the sharpness of the surface. The area per lipid for a vesicle is calculated by dividing the grid surface area *A*_g_ by the number of lipid units in each leaflet: for the outer interface, *A*_L_ = *A*_g_^up^/*n*_up_, and for the inner interface *A*_L_ = *A*_g_^down^/*n*_down_. This functionality can be used to calculate the *A*_L_ and *R*_a_ values for different leaflets as illustrated for a lipid A vesicle (Figure 3).

### Membrane Thickness and Closed Shell Volume

The closed surface membrane thickness (*D*_HH_) and the closed shell volume () are calculated by the *s_shell* routine. For each grid point, the thickness is calculated as the difference between the radial components of the outer and inner surfaces, *D*_HH_(*i*,*j*) = *r*_up_(*i*,*j*) – *r*_down_(*i*,*j*). Therefore, the thickness can be represented in two ways, as a spatial average that evolves over time considering a trajectory of the system or as a temporal average for each grid point of the outer and inner surfaces.

In the spatial average, the *D*_HH_(*i*,*j*) is averaged over all grid points at a specific time frame as
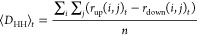
7

Hence, the time evolution of the average thickness  is obtained by collecting its value for every trajectory frame. Likewise, time averages can be calculated for several other properties (*e.g.,*, , , and ), as illustrated in Figure 3. In the temporal average, *D*_HH_(*i*,*j*) is averaged over a time interval with NF trajectory frames for each grid point as
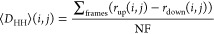
8

**Figure 4:**
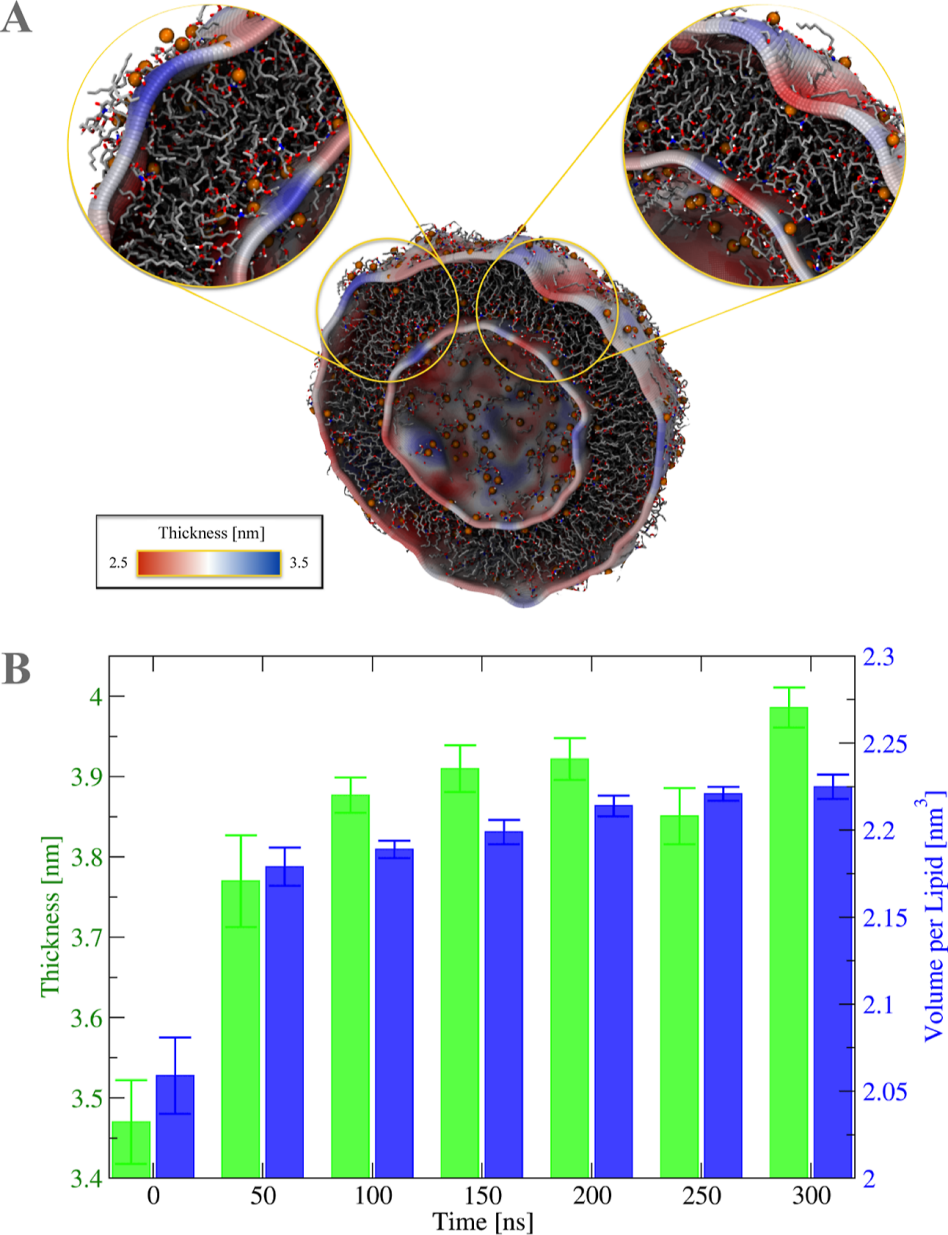
Membrane thickness and volume per lipid. (A) Bidimensional thickness distribution projected on the outer and inner fitted surfaces. Thicker regions are represented in blue and thinner regions are represented in red. (B) Time-averaged thickness and volume per lipid for time intervals of 50 ns. Thicker regions are represented in blue, and thinner regions are represented in red.

The  values are then analyzed using a 3D topographical color map representation in which the grid points in the inner and outer surfaces are colored following a scale of thickness values (Figure 4).

**Figure 5:**
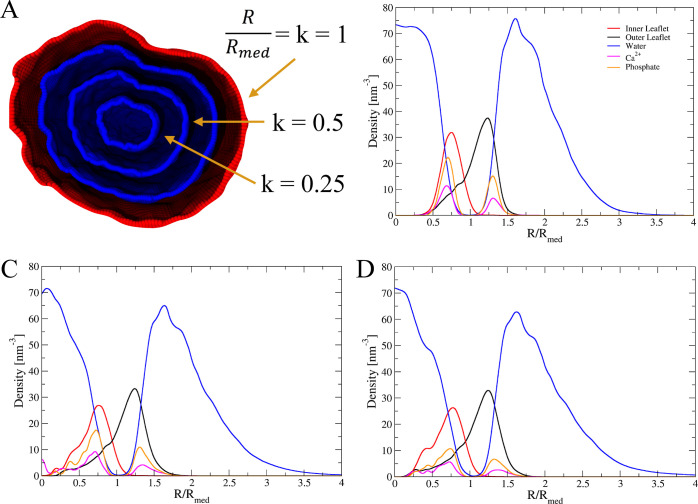
(A) Illustration of the reference surface (red) obtained as the average between the outer and the inner grid surfaces, and the surfaces (blue) generated with different scaling factors *k* < 1. Density profiles for selected chemical groups composing the lipid A vesicle at 0 ns (A), 100 ns (B), and 200 ns (C), respectively. Ca^2+^ and phosphate profiles have been increased by a factor of 5.

There are two different ways to calculate shell volume Δ*V*_sh_ in SuAVE v2.0. One way is calculating the volume between two different grid surfaces that define the two shell limits; for example, the vesicle inner and outer leaflets, . The other way is to calculate the density profile of the system particles where only one grid surface is adopted as a reference surface with average radius *R*_med_ and the shell limits are surfaces with the same shape as the reference but with different average radii generated by a scale factor *k*, , where *k*_sup_ and *k*_inf_ multiply the radial coordinates of all points of the reference grid surface. For systems characterized by two interfaces, such as bilayers and vesicles, the user can select the reference surface as either one or the average surface with the radial coordinates defined as . This average (reference) surface is illustrated in Figure 5D. In both ways, Δ*V*_sh_ is calculated as the difference between the volume comprising the two surfaces with different average radii, , where *R*_sup_ and *R*_inf_ are the average radius of the superior and inferior surfaces that can have different shapes for the case of vesicle membrane volume or the same shape for the density profile shell volumes. Additionally, for vesicles, the volume per lipid is calculated dividing the volume between the outer and inner grid surfaces by the total number of lipid, .

### Number Density Profile

The calculation of density profiles for closed surfaces required a few modifications of the *s_densph* routine previously implemented for open surfaces.^8^ Now, the chemical system is sliced into closed shells centered at *C*_0_ for which the number of particles is counted for each frame of the trajectory. Therefore, the density profiles quantify the density of particles in each shell along the radial direction with the volume of each shell given by Δ*V*_sh_. Once this shell volume is calculated, the density is obtained as the ratio between the number of particles in each shell and the volume of the shell as
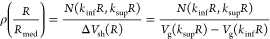
9

The density profiles of different chemical groups of hydrated lipid A vesicles at different time windows are shown in Figure 5. In these profiles, the value *k* = *R*/*R*_med_ = 1 defines the average surface between the inner and outer surfaces (Figure 5A). The density profiles display the water distribution inside the inner leaflet and outside the outer leaflet, the phosphate groups near the two hydrated interfaces, and the Ca^2+^ ions which overlaps with the phosphate distribution.

### Curvature Order Parameter

**Figure 6:**
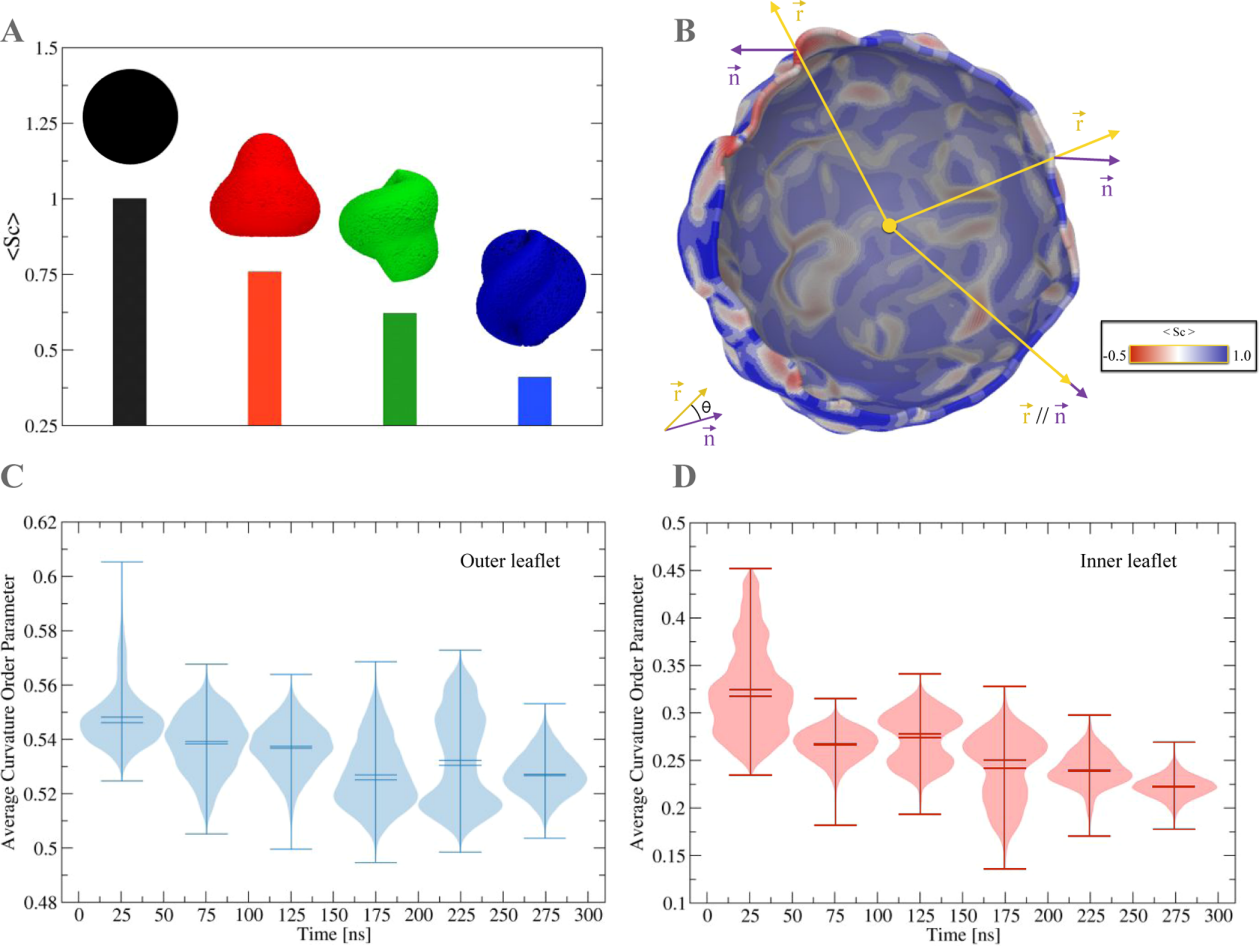
Curvature order parameter *S*_C_. (A) Average *S*_C_ values calculated by SuAVE for four different closed surfaces. (B) S_C_ 3D topological color map on the fitting surface of the lipid A vesicle outer leaflet: regions with deflection angle θ near 90° are shown in red, regions with θ near 0° are shown in blue, and regions with θ around 45° are in white. Radial vector, *r⃗*, and the surface normal vector, *n⃗*, are shown in yellow and purple, respectively. (C,D) Violin plots for the curvature order parameter distribution calculated for the outer and inner leaflets, respectively, along the trajectory evolution.

The curvature order parameter (*S*_C_) is calculated with the *s_bend* routine in the same way as for open chemical surfaces.^8^ However, some minor modifications of the algorithm were required because the system z-axis cannot be used as an alignment reference for the surface normal vector as in the original algorithm. Instead, the surface normal vector *n⃗* is used as an alignment reference for the radial vector *r⃗* of the grid points composing the fitted surface (Figure 2). Hence, a curvature order parameter value of 1.0 means that the chemical interface being fitted by SuAVE v2.0 is a perfect sphere and that the deflection angle θ between the surface normal vector and the radial vector is zero. The curvature order parameter can be represented as average values for different surface shapes (Figure 6A), as 3D topographical color maps in which the vesicle outer surface is colored accordingly to a scale of curvature order parameter values (Figure 6B), and as curvature order parameter distributions for the outer and inner leaflets (Figure 6C,D, respectively).

### Principal Moment of Inertia and Roundness

**Figure 7:**
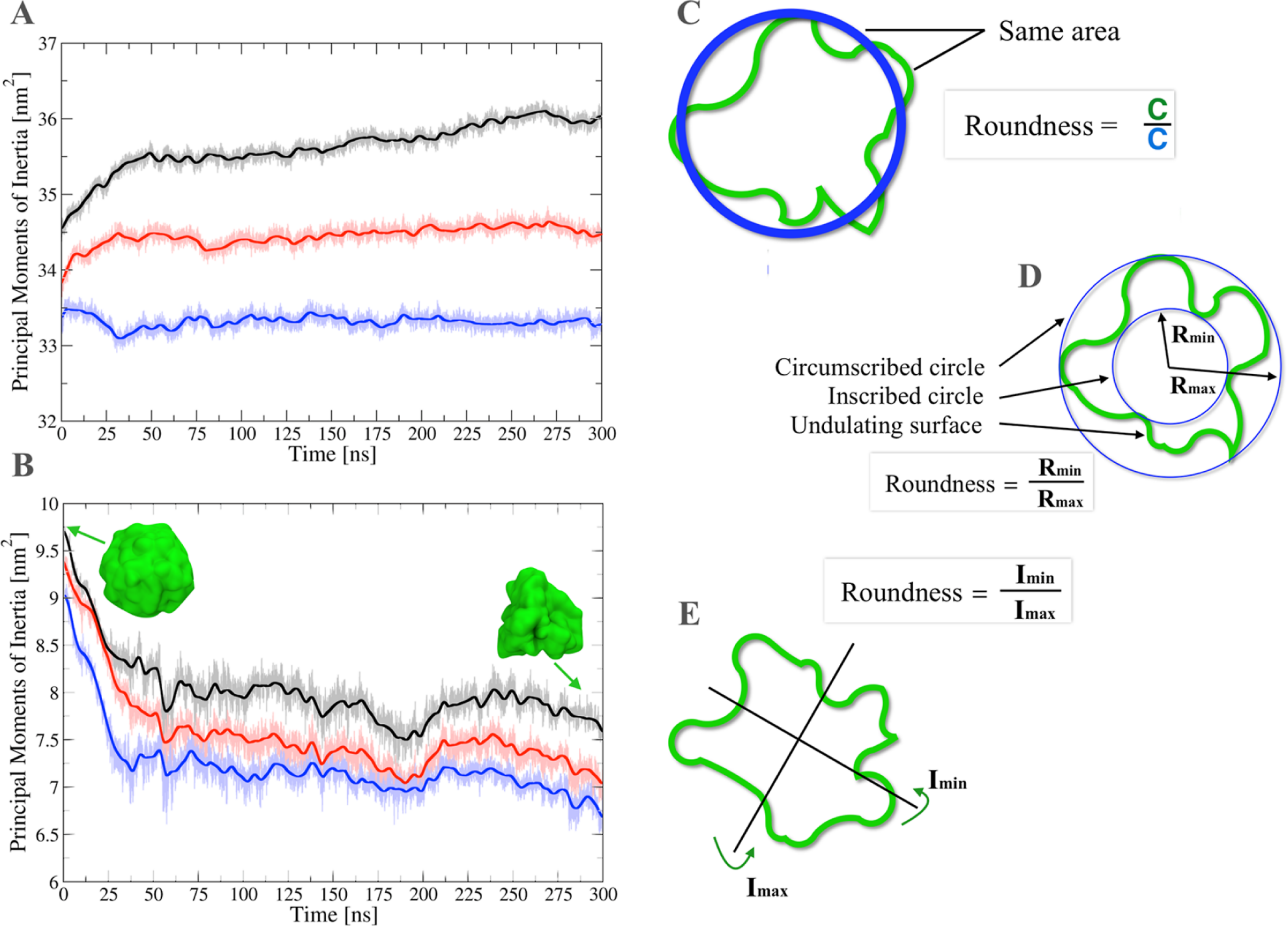
Principal moments of inertia and schematic representations of the three methods implemented in SuAVE 2.0 to calculate the roundness of closed systems. Inertia momenta for the outer (A) and inner (B) leaflet in the lipid A vesicle. The evolving shape of the inner leaflet is illustrated by the fitted grid surface in green. (C) The roundness is calculated as the circumference ratio of the “real” to the ideal spherical topologies, both having the same surface area. (D) The roundness is calculated as the ratio of the largest inner to the smallest outer circle drawn through the center of the vesicle. (E) The roundness is calculated as the ratio between the smallest and the largest moment of inertia where the two lines trespassing the vesicle center correspond to the two the minor *I*_min_ and major *I*_max_ axes of inertia, respectively. In (A,B) discrete Fourier transformation was applied to improve the signal-to noise ratio of the data by using the *s_filter* tool. The shape and volume of porous materials.

The principal moments of inertia and the roundness index are calculated using the *s_inertia* routine of SuAVE (Figure 7). The principal moments of inertia for a closed chemical surface are simply evaluated *via* the diagonalization of the inertia tensor I calculated for the adjusted surface.^16^ This further allows estimating the roundness of the closed surface. The *s_inertia* routine calculates three different expressions of roundness. The first expression is calculated as the ratio of the circumference of a perfectly spherical vesicle to the circumference of the vesicle in consideration with the same area (Figure 7C). The second expression is given by the ratio of the largest inner to the smallest outer concentric circumferences enclosing the vesicle surface (Figure 7D). Finally, the third expression is the ratio of the smallest to the largest inertia moment among the calculated principal moments of inertia of the vesicle (Figure 7E).^17^

Crystalline porous materials have a profusion of industrial applications in gas separation/storage, catalysis, and drug delivery.^18−23^ More recently, this class of highly versatile materials have shown to conduct charge, opening the way for its use as charge storage devices, electrochemical sensors, and electrocatalysts.^24−28^ The most critical feature for each of these applications is the structural property of pore volume for it determines the adsorption, permeability, and storage of guest molecules in porous materials. Hence, the accurate estimate of pore volume is valuable for the identification of potential applications of new crystalline materials. The internal void volume of a porous material is often expressed as the fraction of void volume over the total volume of the material. It can be calculated from the crystal structure and can be experimentally estimated *via* measurements of nitrogen uptake under controlled conditions of temperature and pressure. Whenever X-ray structures of metal–organic frameworks (MOFs) are available, it is useful to compare computational and experimentally derived volumes to assess microscopic information not readily available from experiments.

**Figure 8:**
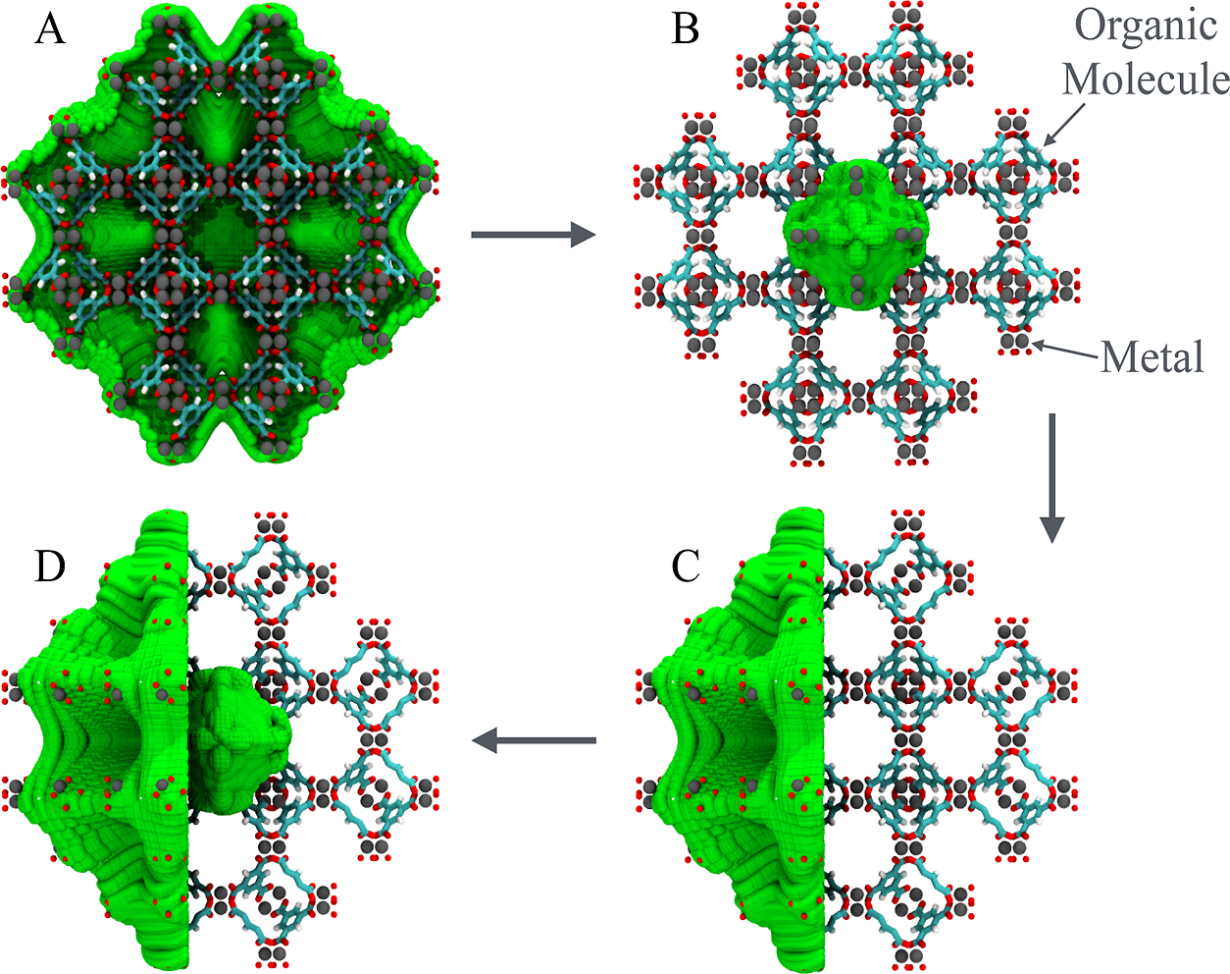
Representation of complex interfaces of porous materials. The grid surface was fitted to the external interface and inner pore of the HKUST-1 MOF. Frontal (A,B) and lateral (C,D) views of the constructed grid fitted on the external interface and inner pore respectively. The X-ray structure of HKUST-1 is shown as a 2 × 2 × 2 cell with the grid surface in green, imidazolate linkers in sticks, and zinc atoms in gray spheres.

SuAVE calculates several structural properties of relevance for crystalline porous materials such as pore diameter (*D*_P_), pore volume (*V*_P_), pore area (*A*_P_), the total void volume (*V*_T_) of the unit cell structure, and the void fraction θ_Gm_ (Table 1). This latter quantity can be compared to the experimentally measured pore volumes from nitrogen isotherms. The structural properties are calculated using the *s_pore* routine as follows: first, a grid-fitted surface is built based on the positions of atoms that encompass the porous material structure in the unit cell (Figure 8A). The surface is calculated *via* a modification of the weight function expressed by
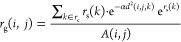
10where  is the weight normalization and *α* is the width of the Gaussian distribution described by eq 2 with the same β and γ parameters.

**Table 1:** Structural Properties Calculated by SuAVE for Selected Crystalline Porous materials^29^^a^

MOF	*D*_P_	*V*_P_	*A*_P_	sphericity	*V*_T_	θ_Gm_	θ_Expt_
HKUST-1	1.3414	1.2254	7.9358	0.6978	12.2098	0.671	0.643
DUT-13	2.4077	6.7153	26.6766	0.6453	64.1284	0.840	0.762
NU-125	2.5363	7.9616	29.5624	0.6522	33.3237	0.755	0.746
PCN-46	1.4381	1.2751	7.9034	0.7195	10.2267	0.742	0.626
PCN-61	1.2211	7.1344	26.6808	0.6717	60.1102	0.764	0.762
SNU-30	1.5624	2.4216	12.1711	0.7165	11.9408	0.833	0.107
SNU-50	1.5969	1.6941	9.1961	0.7473	11.7666	0.669	0.702
UTSA-20	1.3945	1.2122	7.5694	0.7264	4.3192	0.655	0.573
UTSA-34	0.9868	0.4744	3.9578	0.7432	9.3690	0.649	0.455
UTSA-62	1.7936	2.7341	14.8666	0.6360	8.8131	0.798	0.537

a *D*_P_ is the diameter (nm) of the largest pore, *V*_P_ and *A*_P_ are the internal void volume (nm^3^) and total area (nm^2^) of the largest pore, respectively, *V*_T_ is the total void volume (nm^3^) of the unit cell structure, and θ_Gm_ is the void fraction calculated by the geometric pore volume methodology (see ref (24)) with *N* = 500,000. θ_Expt_ values were calculated from the experimental measurements of the free volume and density of the materials according to eq S1 and Table S6 in the Supporting Information.

In our implementation, the void volume is calculated by randomly displacing points in the cell unit but avoiding overlap with the atoms in the crystal cell unit. This is performed *via* the assignment of the distances between each sampling point and the nearest atom in the interface surrounding the pore. These atoms are represented by atom-specific van de Waals radii (*r*_vdW_) so that when the distance between a given point and the atom is smaller than half of its van de Waals radius, a value of 0 is assigned to the point. Otherwise, a value of 1 is assigned. Therefore, a Heaviside function is used,  = 0 for  and  = 1 for . The pore void volume is calculated using eq 5 (Figure 8B–D) and the void volume fraction is given by^29^
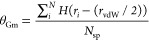
11where *N*_sp_ is the number of sampling points used for the assessment of the void fraction.

Ongari and co-workers performed a systematic evaluation of the accuracy and discrepancies of a number of computational techniques to calculate pore volume from crystalline structures of MOFs.^29^ It was shown that each method computes slightly different portions of the internal volume due to different definitions of the probe used to estimate the pore volume. The method implemented in SuAVE v.2 is conceptually an extension of the geometric pore volume method^29^ which combines good accuracy with easiness of implementation within the SuAVE framework. However, in our implementation, the pore volume is sampled by randomly displacing points in the unit cell while the implementation discussed in ref (29) is based on a uniform distribution of sampling points on a grid with a 0.2 Å bin size. One important feature of the Gm algorithm implemented in SuAVE is that it converges within less than 0.01% of the void fraction with only 6.84 points per cubic angstrom (Figure S5).

**Figure 9:**
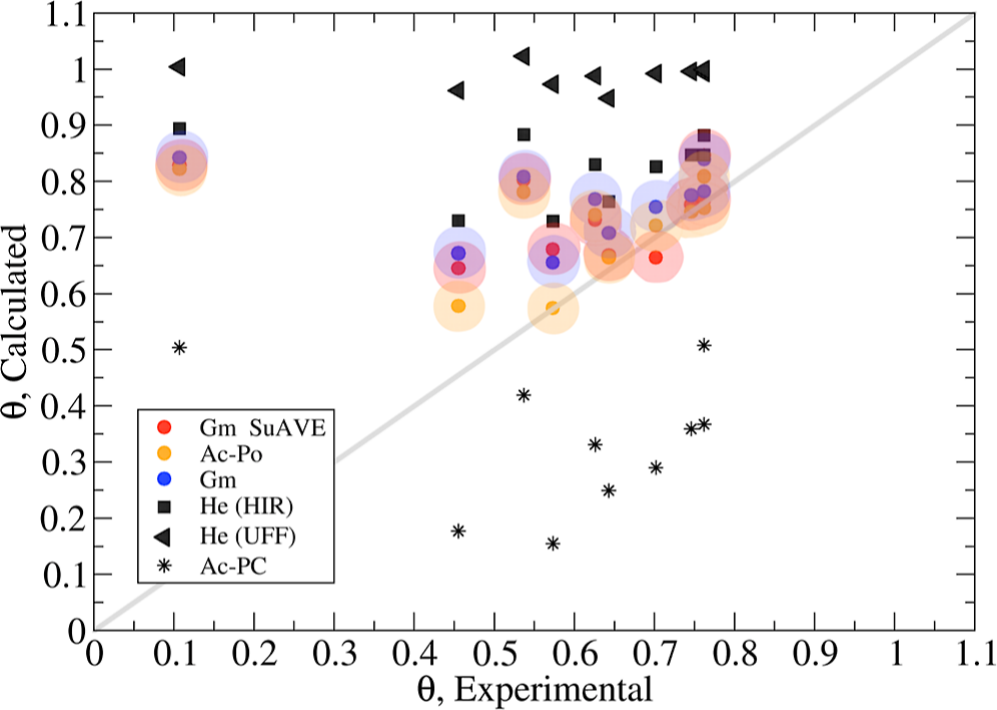
Comparison of calculated and experimentally derived porosities θ for the benchmark set of structures presented in Table 1. The methods used to calculate the porosities are designed accordingly to geometric pore volume (Gm), accessible probe-occupiable pore volume (Ac-PO), accessible probe center pore volume (Ac-PC), helium pore volume with van der Waals atomic parameters for He and all atoms taken from the Universal force field^40^ (He-UFF), and from Hirschfelder^41^ (He-Hir). Experimental values were calculated from the experimental measurements of the free volume and density of the materials according to eq S1 and Table S6 in the Supporting Information.

A comparison of void fraction values calculated with the geometric pore volume implementation in SuAVE and the best performing methods assessed in ref (29) against the experimental values for a benchmark set of 10 MOFs^30−39^ is presented (Figure 9). For simplicity, we designate the different methods using the same acronyms in ref (29), that is, geometric pore volume (Gm), accessible probe-occupiable pore volume (Ac-PO), accessible probe center pore volume (Ac-PC), the helium pore volume with van der Waals parameters for the framework and for helium taken from the Universal force field^40^ (He-UFF), and from Hirschfelder^41^ (He-Hir). A complete description of the various methods is provided in ref (29).

The comparison of calculated and experimental porosity values indicate that the geometric pore volume (Gm) and the accessible probe-occupiable pore volume (Ac-PO) methods exhibits the best performance (Figure 9). The deficiencies of the remaining approaches have already been discussed elsewhere.^29^ The two implementations of the Gm approach exhibit a correlation of 91% and reproduce the experimental values with an average root-mean-square error of 0.0799 (SuAVE) and 0.0792 (ref (29)). The Gm and Ac-PO approaches exhibit a greater prediction accuracy for larger experimental values of porosity (Figure 9). Therefore, it is important to understand the source of discrepancies between calculated and experimental quantities for the frameworks with small experimental porosity values and more specifically for SNU-30 (Table 1).

Experimental measurements of porosity rely on the loading of nitrogen gas in the pores of the MOF. Although N_2_ has a small size and should only interact weakly with the framework, experimental values cannot account for small regions where the N_2_ molecule cannot fit (*e.g.*, small interstices between atoms, pores connected by narrow channels inaccessible to N_2_ molecules). Furthermore, incomplete solvent removal and structure shrinking are common during the synthesis and characterization of MOFs.^42,43^ MOFs are manufactured *via* solvothermal synthesis in polar solvents with high boiling point so that the measurement of internal surface area and porosity requires the removal of solvent and guest molecules from the framework pores *via* a process termed activation. Frequently, postactivation MOFs exhibit lower-than-predicted surface areas and broadened powder X-ray diffraction peaks distinctive of decreased framework crystallinity.^43,44^ A large difference between experimental and computed surface areas for SNU-30 has already been attributed to postactivation pore collapse in the original publication reporting on its synthesis. It is also notable that all tested algorithms predict a larger porosity than the experimental estimate (Figure 9). Likewise, incomplete solvent removal and structure shrinking can also explain the smaller discrepancies between calculated and experimental estimates for UTSA-34 and UTSA-62.^29^

## Data and Software Availability

SuAVE is a free, open-source software licensed under the GNU General Public License v2 or later. SuAVE has been developed using version control, unit testing, and continuous integration. A documented API with examples of how to use each analysis tool is available at https://github.com/SuAVE-Software/source. The current state of code development and planned implementations are also available at the GitHub website. SuAVE follows GitHub guidelines with the development of the main code directly from the master branch and immediate release of new versions after the addition of new functionalities or corrections. Feature requests and code issues can be submitted *via* GitHub. For inquiries and comments, please send an email to suave.biomat@gmail.com.

## Supporting Information Available

Parameterization and validation of β and γ for the accurate interpolation of points across complex closed surfaces, assessment of convergence and computing time to interpolate surface points onto the chemical interface as a function of the grid density, and values of the MOF porosities calculated through the free volume and density of the materials (PDF)
