# Supplementary material for: Surface Assessment via Grid Evaluation (SuAVE) for Every Surface Curvature and Cavity Shape
Source: J Chem Inf Model. 2022 Aug 10;62(19):4690–701. doi: 10.1021/acs.jcim.2c00673 (PMC9554907; doi:10.1021/acs.jcim.2c00673)
Supplement: Supplementary file 1 — ci2c00673_si_001.pdf [file ci2c00673_si_001.pdf]

Supporting Information

Surface Assessment via Grid Evaluation (SuAVE) for Every Surface Curvature and Cavity Shape

*Denys E. S. Santos†, Kaline Coutinho§ and Thereza A. Soares§¶**

†Dept. of Fundamental Chemistry, Federal University of Pernambuco, Cidade Universitária, Recife 50740-560, Brazil

§ Instituto de Física, Universidade de São Paulo, Cidade Universitária, São Paulo 05508-090, Brazil

¶ Hylleraas Centre for Quantum Molecular Sciences, University of Oslo, 0315 Oslo, Norway

Corresponding author e-mail address: thereza.soares@usp.br

The fitting parameter α

The evaluation of the fitting parameter used in the interpolation process followed the same protocol as described for open surfaces.[1] The influence of this parameter was observed through an essay with four different closed surfaces (r_1, r_2, r_3 and r_4) whose format can efficiently extrapolate vesicle and micelle structures, *i.e.*, the chosen surfaces may not be observed in reality, however, they are useful to prepare the code to interpolate closed surfaces in a plethora of morphologies. The radial component of the surface defined on spherical coordinates, is a function of θ and φ angle as can be seen bellow.

$$r_1(\theta,\varphi) = C + cos\,(2\varphi) \qquad 0 \le \varphi \le \pi,\;\; 0 \le \theta < 2\pi \qquad (SI-1a)$$

$$r_2(\theta,\varphi) = C + cos\,(3\varphi\; +\; \theta) \qquad 0 \le \varphi \le \pi,\;\; 0 \le \theta < 2\pi \qquad (SI-1b)$$

$$r_3(\theta,\varphi) = C + cos\,(2\varphi\; +\; 3\theta) \qquad 0 \le \varphi \le \pi,\;\; 0 \le \theta < 2\pi \qquad (SI-1c)$$

$$r_4(\theta,\varphi) = C + sin\,(4\varphi)cos\,(2\theta) \qquad 0 \le \varphi \le \pi,\;\; 0 \le \theta < 2\pi \qquad (SI-1d)$$

In order to allow distinct surface densities on the evaluation of the parameter α, the constant C was defined by two values, namely 4 and 5, composing the Set 1 and Set 2. These values were chosen together with the number of sampling points used during the essays to generate surfaces with densities of sampling points ranging from 0.1 points/nm^2 up to 13.0 points/nm^2, typical values observed for lipid vesicles or micelles. Using the four closed surfaces (r_1, r_2, r_3 and r_4) and the range established for the density of sampling points it is possible to evaluate the effect of the fitting parameter as to the RMSD between the fitting GRID and the sampling surface. Figure S1 exemplifies the result of this benchmark for the surface r_1. The values of the fitting parameter that minimized the RMSD, as observed in Figure S1, define the relation between this parameter and the density of sampling points.

Tables S1, S2, S3 and S4 show the relation between the fitting parameter and the density of sampling points for the surfaces r_1, r_2, r_3 and r_4.

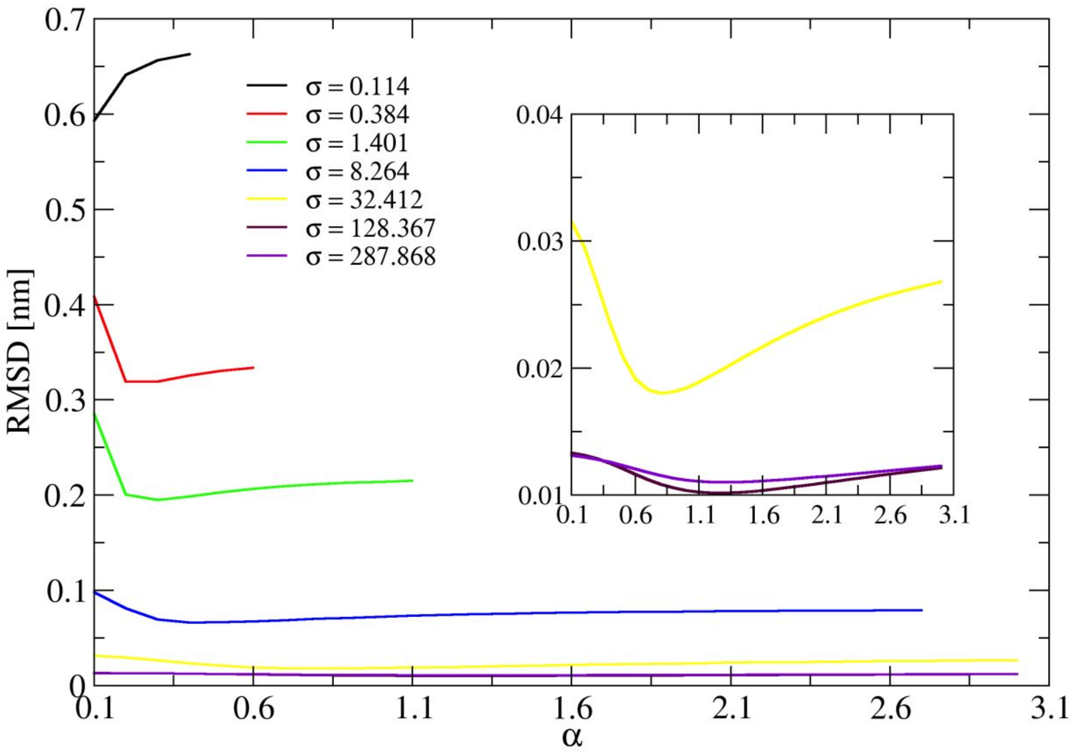

Figure S1. RMSD between the developed interpolant GRID and the surface points for the function r_1.

Table S1: Values for α parameter defined for each density of sampling points $r_1(\theta,\varphi)$

Points	Area (nm²)	σ (points/nm²)	ln(σ)	α	RMSD (nm)
		**Set 1 (C = 4)**			
36	313.632	0.115	-2.165	0.100	0.593
121	313.632	0.386	-0.952	0.200	0.319
441	313.632	1.406	0.341	0.300	0.195
2601	313.632	8.293	2.115	0.400	0.066
		**Set 2 (C = 5)**			
36	201.521	0.179	-1.722	0.100	0.577
121	201.521	0.600	-0.510	0.300	0.311
441	201.521	2.188	0.783	0.400	0.180
2601	201.521	12.907	2.558	0.600	0.059

Table S2: Values for α parameter defined for each density of sampling points $r_2(\theta,\varphi)$

**Points**	**Area** (nm²)	**σ** (points/nm²)	**ln(σ)**	**α**	**RMSD** (nm)
		**Set 1 (C = 4)**			
36	314.763	0.114	-2.168	0.200	0.706
121	314.763	0.384	-0.956	0.200	0.384
441	314.763	1.401	0.337	0.300	0.289
2601	314.763	8.263	2.112	0.800	0.189
		**Set 2 (C = 5)**			
36	201.731	0.178	-1.723	0.200	0.662
121	201.731	0.600	-0.511	0.300	0.387
441	201.731	2.186	0.782	0.400	0.285
2601	201.731	12.893	2.557	1.000	0.188

Table S3: Values for α parameter defined for each density of sampling points $r_3(\theta,\varphi)$

**Points**	**Area** (nm²)	**σ** (points/nm²)	**ln(σ)**	**α**	**RMSD** (nm)
		**Set 1 (C = 4)**			
36	315.004	0.114	-2.169	0.100	0.780
121	315.004	0.384	-0.957	0.200	0.519
441	315.004	1.400	0.336	0.500	0.424
2601	315.004	8.257	2.111	1.800	0.219
		**Set 2 (C = 5)**			
36	201.545	0.179	-1.722	0.100	0.746
121	201.545	0.600	-0.510	0.300	0.524
441	201.545	2.188	0.783	0.600	0.426
2601	201.545	12.905	2.558	2.500	0.221

Table S4: Values for α parameter defined for each density of sampling points $r_4(\theta,\varphi)$

Points	Area (nm²)	σ (points/nm²)	ln(σ)	α	RMSD (nm)
		**Set 1 (C = 4)**			
36	314.731	0.114	-2.168	0.100	0.669
121	314.731	0.384	-0.956	0.300	0.313
441	314.731	1.401	0.337	0.400	0.226
2601	314.731	8.264	2.112	0.900	0.088
		**Set 2 (C = 5)**			
36	200.647	0.179	-1.718	0.100	0.623
121	200.647	0.603	-0.506	0.400	0.311
441	200.647	2.198	0.787	0.500	0.214
2601	200.647	12.963	2.562	1.100	0.082

The resulting profile for the α parameter is obtained as the average of the preceding tables.

Table S5: Average values for α, defined for each density of sampling points.

σ (points/nm²)	ln(σ)	α	ln(α)
0.114	-2.168	0.125	-2.079
0.385	-0.955	0.225	-1.492
1.402	0.338	0.375	-0.981
8.269	2.113	0.975	-0.025
0.179	-1.722	0.125	-2.079
0.601	-0.509	0.325	-1.124
2.190	0.784	0.475	-0.744
12.917	2.559	1.300	0.262

The data encompassed on Table S5 can be easily depicted on Figure S2, besides the best relationship between the sampling point density and the fitting parameter α (Equation S2).

$$\alpha = 0.0382\sigma^{0.9968} \qquad (S2)$$

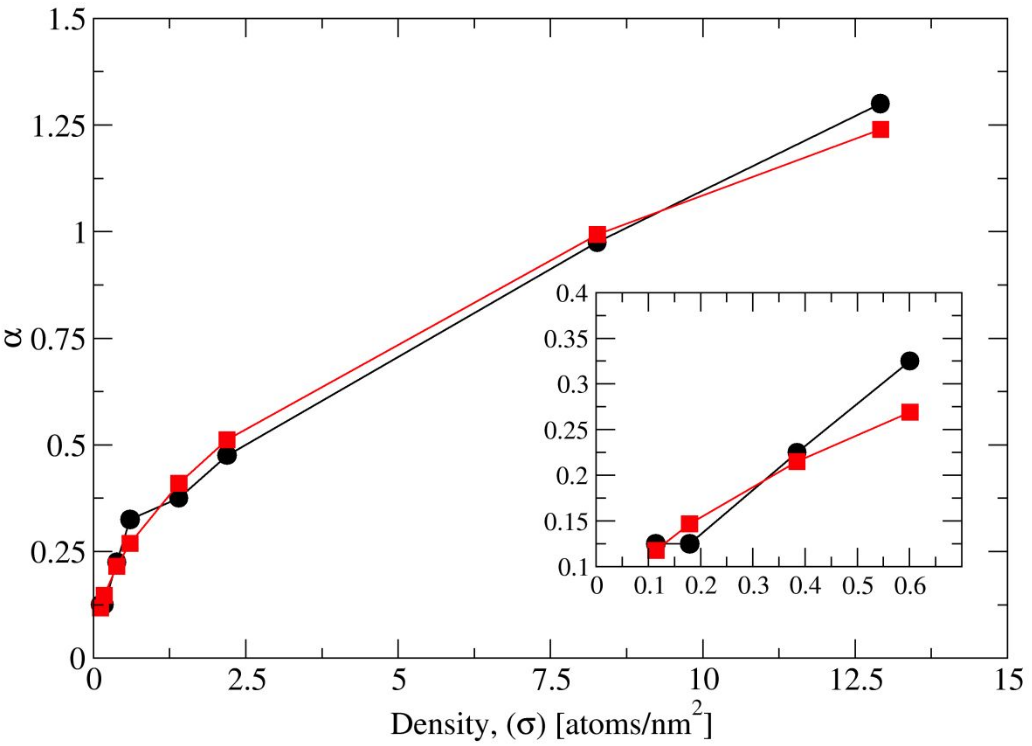

Figure S2: Fitting parameter α as a function of the density of sampling points. The points in black represent the data presented in Table S5, while the squares in red were obtained from the model in Equation S2 that explains the dependency of these parameters.

Although the chosen values for the fitting parameter provide the minimum RMSD values for each density of points, low densities are not able to describe the morphology of the surfaces adequately. Thus, at this condition, the obtained RMSD values are still high and the calculated surface may not describe optimally the chemical interface being sampled. In order to establish a minimum number of surface sampling points to ensure that the calculated surface fully represents the chemical interface, it suffices to evaluate density values that, independent of the fitting parameter, promote RMSD values equal or less than 2.0 nm.

In Figure S3, it is possible to observe the complete lack of connection between the two calculated surfaces with the lowest point densities compared to the original surface (blue surface). This problem is corrected upon the increase in the surface point density to values of at least 1.400 points/nm^2, which corresponds to a RMSD of 0.424 nm between the original and adjusted surfaces (Figure S3). Full convergence is reached for point densities of 8.257 points/nm^2, leading to RMSD of 0.219 nm between the original and adjusted surfaces (Table S3)

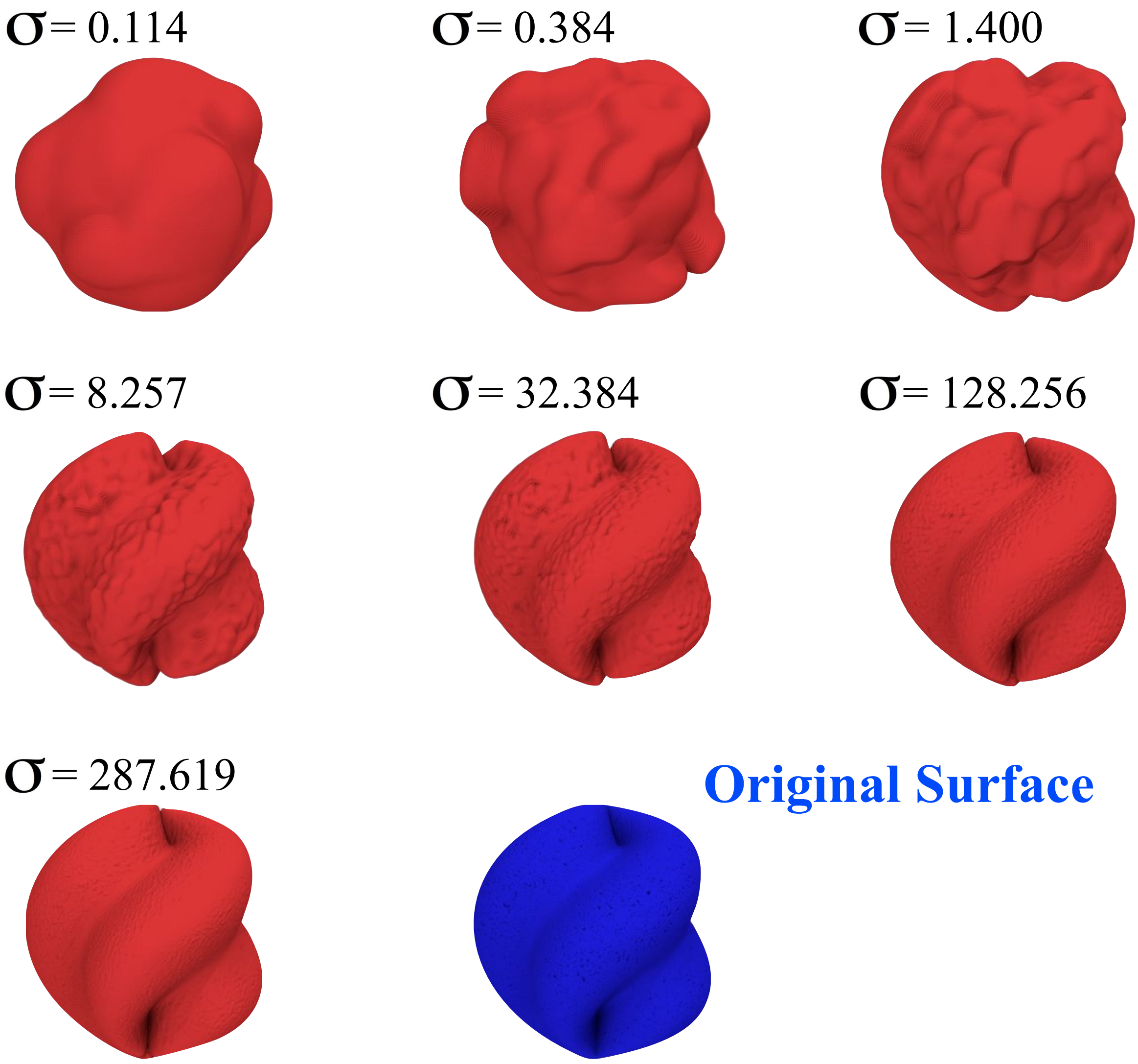

Figure S3. Graphical representation of the fitting of the calculated surface (red), when using a mesh of 90601 points to interpolate different density of points of the function r_3.

Property convergence and computer processing time to interpolate calculated surface onto chemical surface

A convergence analysis has been carried out to evaluate how the properties calculated by SuAVE change as we improve the number of GRID partitions, and in its turn, the GRID refinement. Figure S4 shows how the total area varies in respect to the GRID refinement. In the same figure it is possible assess the elapsed time required by the computer to finish this analysis for a set of structures consisting of the 50 initial frames of the trajectory analyzed in the paper.

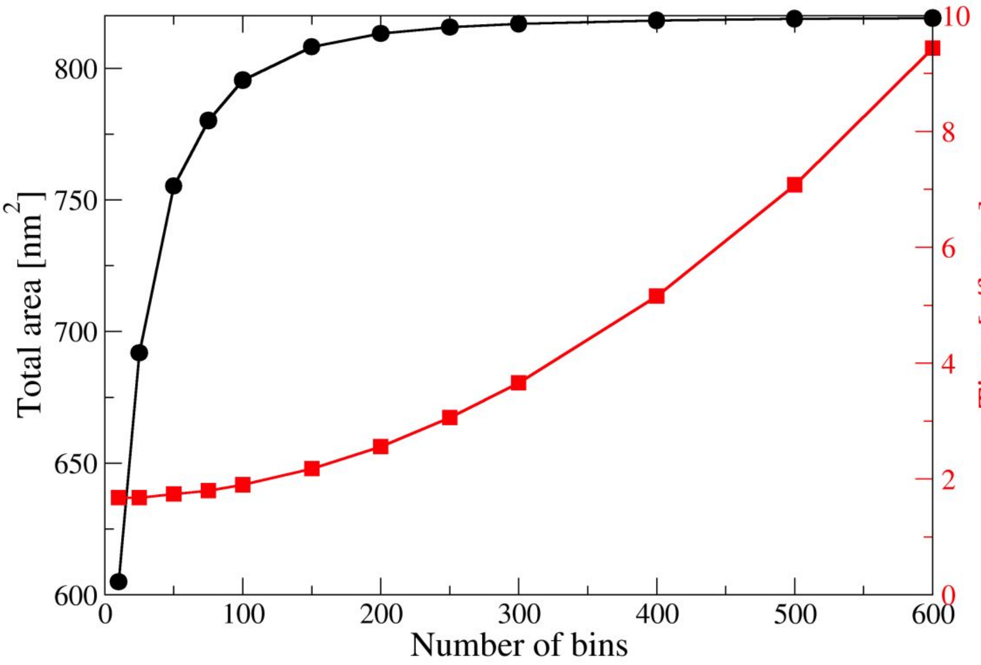

Figure S4. Convergence of the area versus number of bins and the computer processing time per frame required to interpolate the calculated surface onto chemical surface versus the number of bins. Each bin is a rectangular partition along the φ- and θ-angle.

The larger the number of bins, the higher the GRID refinement and the processing time to calculate and adjust the calculated surface onto the chemical interface. It is clearly not advantageous to increase the number of bins beyond the value of 200 for the current systems or larger ones, corresponding to a mesh resolution of about of 20301 points.

Experimental data for the calculation of porosity

The experimental values of the MOF porosities have been calculated through the free volume and density of the materials according to the following equation.

$$\theta = \upsilon_{pore}\rho_{crystal} \quad \text{eq. S1}$$

where υ_{pore} is commonly expressed in cubic centimeters per gram of crystal.

Table S6. Description of experimental values of free volume and density of MOF crystals. Data from ref.[2]

MOF	υ_{pore} [$cm^3.g^{-1}$]	$\rho_{crystal}$ [$g.cm^{-3}$]	θ
**HKUST-1**	0.730	0.881	0.643
**DUT-13**	1.980	0.385	0.762
**NU-125**	1.290	0.578	0.746
**PCN-46**	1.012	0.619	0.626
**PCN-61**	1.360	0.560	0.762
**SNU-30**	0.280	0.381	0.107
**SNU-50**	1.080	0.650	0.702
**UTSA-20**	0.630	0.910	0.573
**UTSA-34**	0.542	0.840	0.455
**UTSA-62**	0.910	0.590	0.537

1. Santos, D. E. S.; Pontes, F. J. S.; Lins, R. D.; Coutinho, K.; Soares, T. A. Suave: A Tool for Analyzing Curvature-Dependent Properties in Chemical Interfaces. J Chem Inf Model 2020, 60, 473-484.
2. Mason, J. A.; Veenstra, M.; Long, J. R. Evaluating Metal–Organic Frameworks for Natural Gas Storage. *Chemical Science* **2014**, 5, 32-51
